# Overexpression of FOXO3, MYD88, and GAPDH Identified by Suppression Subtractive Hybridization in Esophageal Cancer Is Associated with Autophagy

**DOI:** 10.1155/2014/185035

**Published:** 2014-01-08

**Authors:** Mohammad Soltany-Rezaee-Rad, Negar Mottaghi-Dastjerdi, Neda Setayesh, Gholamreza Roshandel, Farzaneh Ebrahimifard, Zargham Sepehrizadeh

**Affiliations:** ^1^Department of Pharmaceutical Biotechnology and Pharmaceutical Biotechnology Research Center, School of Pharmacy, Tehran University of Medical Sciences, Tehran 1417614411, Iran; ^2^Pharmaceutical Sciences Research Center, Sari School of Pharmacy, Mazandaran University of Medical Sciences, Sari 48175-861, Iran; ^3^Golestan Research Center of Gastroenterology and Hepatology, Golestan University of Medical Sciences, Golestan 49186-619, Iran; ^4^Department of General Surgery, School of Medicine, Shahid Beheshti University of Medical Sciences, Tehran 19857-17443, Iran

## Abstract

To find genes involved in tumorigenesis and the development of esophageal cancer, the suppression subtractive hybridization (SSH) method was used to identify genes that are overexpressed in esophageal cancer tissues compared to normal esophageal tissues. In our SSH library, the forkhead box O3 (FOXO3), glyceraldehyde-3-phosphate dehydrogenase (GAPDH), and myeloid differentiation primary response 88 (MYD88) genes were the most highly upregulated genes, and they were selected for further studies because of their potential role in the induction of autophagy. Upregulation of these genes was also observed in clinical samples using qRT-PCR. In addition, coexpression analysis of the autophagy-related genes Beclin1, ATG12, Gabarapl, PIK3C3, and LC3 demonstrated a significant correlation between the differentially overexpressed genes and autophagy. Autophagy is an important mechanism in tumorigenesis and the development of chemoresistance in cancer cells. The upregulation of FOXO3, GAPDH, and MYD88 variants in esophageal cancer suggests a role for autophagy and provides new insight into the biology of esophageal cancer. We propose that FOXO3, GAPDH, and MYD88 are novel targets for combating autophagy in esophageal cancer.

## 1. Introduction 

Esophageal cancer is one of the most aggressive and life-threatening types of carcinoma in developing countries, and it has a high incidence rate in some geographical regions, particularly in the “Asian esophageal cancer belt,” which extends from the Caspian Littoral in Iran, Turkmenistan, Uzbekistan, and Kazakhstan to the northern provinces of China [1]. Esophageal cancer is among the top 10 causes of cancer-related deaths worldwide [2]. Although some altered oncogenes and tumor suppressor genes have been identified in esophageal cancer (e.g., p53 deletion, p21 alteration, and amplification of CCND1 and c-myc), the fundamental molecular mechanisms leading to esophageal cancer remain unknown [3–6]. The identification of genes that are differentially expressed in esophageal cancer cells allows for the identification of new biomarkers and therapeutic target genes. In addition, this strategy could lead to an improved understanding of the molecular biology and mechanisms of carcinogenesis in esophageal cancer.

In contrast to apoptosis, autophagy is primarily a cell survival process; thus, autophagy has been considered an important mechanism in chemoresistance and is known as a survival factor for tumor cells in the early stages of tumorigenesis [7–10]. In this study, suppression subtractive hybridization (SSH) was used to identify genes that are overexpressed in esophageal cancer cells. Among the identified ESTs from the constructed SSH library, potential autophagy-inducing genes (FOXO3, MYD88, and GAPDH) were selected for further analysis. The incidence of autophagy was also determined in clinical tissue samples by quantifying autophagy-related/regulatory genes. This study provides new information concerning the genes associated with the development of esophageal cancer, particularly those involved in autophagy, which could have a significant influence on the diagnosis and treatment of this type of cancer, which typically has a poor prognosis.

## 2. Materials and Methods

### 2.1. Tissue Collection

The samples used for the SSH were surgically resected using esophagectomy from a patient prior to chemotherapy. Normal tissue was resected 10 cm far from the tumor. A pathologist dissected the target tissues under the microscope with special care for minimal contamination of nonepithelial cells. For confirmation of overexpressed genes by qRT-PCR, 10 samples from 5 patients (5 tumors and 5 normal tissues from the same patients) were collected using endoscopy. Target tissues were immersed in 10 volumes of an RNAlater solution (Ambion, Austin, TX, USA). The samples were stored at −80°C freezer. The resected tissues were examined using hematoxylin-eosin (H&E) staining. The consent form was approved by the Biologic Sampling Ethics Committee of the Tehran University of Medical Sciences and obtained from patients prior to sampling.

### 2.2. Extraction of Total RNA

Tissues were lysed using a mortar and pestle and liquid nitrogen. Two mL of Tripure isolation reagent (Roche Applied Science, Indianapolis, IN, USA) was added. The lysed tissues were passed through a 20-gauge needle 10 times for homogenization. The procedure was performed according to the manufacturer's instructions. The concentration and purity of the total RNA were determined using a BioPhotometer (Eppendorf, Hamburg, Germany). RNA quality was assessed using a 1% denatured agarose gel.

### 2.3. Isolation of mRNA

mRNA was isolated using the DynaBeads mRNA Isolation Kit (Dynal, Lake Success, NY, USA). Briefly, DynaBeads oligo (dT)_25_ were equilibrated with 100 *μ*L of binding buffer (100 mM Tris-HCl, 500 nM LiCl, 10 mM EDTA, 1% LiDS, and 5 mM DTT). The extracted total RNA was diluted to 100 *μ*L in binding buffer, and the equilibrated DynaBeads were added and incubated for 5 min at 37°C in a shaking incubator. The beads were then placed on a magnet, the supernatant was removed, and the beads were washed twice using 200 *μ*L of washing buffer (10 mM Tris-HCl, 0.15 M LiCl, and 1 mM of EDTA). Elution buffer (10 *μ*L) was added to the beads prior to incubation at 65°C for 2 min. The beads were then immediately placed on the magnet, and the eluted mRNA was isolated. To improve the reduction of background rRNA, this protocol was repeated on the eluted mRNA. The purity of the purified mRNA was assessed using a 1% denatured agarose gel.

### 2.4. Construction of the SSH Library

When constructing a subtracted library, it is routine procedure to use a single sample pair. SSH library construction was achieved using the PCR-Select cDNA subtraction kit (Clontech, Palo alto, CA, USA) according to the manufacturer's instructions. Briefly, 2 *μ*g mRNA from normal (as Driver) and tumor (as Tester) tissues was applied for the first and second cDNA synthesis. The resulting cDNA was digested with *Rsa*I, and the blunt-ended double-stranded cDNA was purified. The Tester cDNA was divided into two parts, which were individually ligated to adaptor 1 and adaptor 2. The adaptor 1- and adaptor 2-ligated cDNAs were separately hybridized with excess amounts of Driver cDNA for 8 hr at 68°C. The two hybridization mixtures were combined and hybridized with excess amounts of Driver cDNA overnight at 68°C. The final hybridization mixture was diluted using 200 *μ*L of dilution buffer for two subsequent rounds of PCR. The constructed SSH libraries were confirmed using 1% agarose gel electrophoresis.

### 2.5. Cloning and Similarity Searches

The constructed SSH libraries were purified using the PCR Product Purification Kit (Roche Applied Science), and the purified PCR products were ligated into the pUC19 cloning vector and transformed into *Escherichia coli* NovaBlue competent cells (Novagen, Madison, WI, USA). Positive clones were verified using the colony PCR method with the adaptor-specific primers N1 (5′-TCGAGCGGCCGCCCGGGCAGGT-3′) and N2R (5′-AGCGTGGTCGCGGCCGAGGT-3′), and the plasmids were isolated for sequencing using the High Pure Plasmid Isolation Kit (Roche Applied Sciences). Single direction DNA sequencing was performed using the BigDye terminator v3.1 sequencing kit and a 3730xl automated sequencer (Applied Biosystems, Foster City, CA, USA). Similarity searches were performed using the BLASTn, tBLASTn, and tBLASTx algorithms in the NCBI GenBank databases (http://blast.ncbi.nlm.nih.gov/Blast.cgi) to analyze the sequences.

### 2.6. Analysis of the Subtraction Efficiency

The efficiency of the subtraction method was analyzed by comparing the abundance of a nondifferentially expressed housekeeping gene (e.g., beta actin) before and after subtraction. Briefly, the subtracted and nonsubtracted samples were diluted 10-fold in H_2_O as a template for PCR. Real-time PCR reactions were prepared by adding 10 *μ*L of SYBR Premix Ex Taq (Takara, Kusatsu, Japan), 1 *μ*L of sample, 0.8 pmol of the beta actin primers, 0.4 *μ*L of ROX dye, and DEPC-treated water to a final volume of 20 *μ*L. The thermal cycling parameters for the reaction consisted of an initial heating of 10 min at 95°C, followed by 40 cycles of 30 sec at 95°C and 1 min at 60°C. Melting curve analysis was performed by increasing the temperature from 65°C to 95°C in 0.1°C/second increments for each fluorescence reading using the Step-One-Plus apparatus (Applied Biosystems). The subtraction efficiency was determined using the relative expression of the beta actin gene in the subtracted and nonsubtracted samples.

### 2.7. qRT-PCR Analysis of the FOXO3, MYD88 Variants, and GAPDH Genes

**Figure 1:**
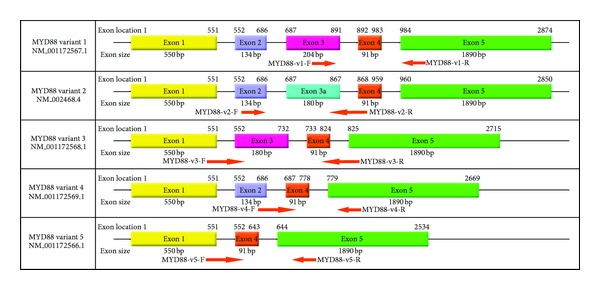
Alternative splicing variants of the MYD88 gene and designed primers locations.

To analyze the overexpression of the genes identified using the SSH, normal and cancerous endoscopic tissue samples were collected from patients. Total RNA was extracted using the Tripure Isolation Reagent (Roche Applied Sciences). One *μ*g of total RNA was used for cDNA synthesis with the Expand Reverse Transcriptase (Roche Applied Sciences) and oligo (dT)_18_ primer.  Table 1 presents the primers that were designed to amplify the FOXO3, GAPDH, and beta actin genes in the qRT-PCR reaction. Alternative splicing of MYD88 variants was analyzed using the real-time PCR method. In this method, primers were designed based on variant-specific exons from MYD88. The designed primers locations for the specific MyD88 variants are depicted in Figure 1. The primers were designed using PrimerSelect software version 7.1.0 (DNAstar, Madison, WI, USA) and synthesized by the TAG Company (TAG Copenhagen, Copenhagen, Denmark). The qRT-PCR reaction was prepared using 10 *μ*L of SYBR Premix Ex Taq (Takara), 2 *μ*L of cDNA, 0.8 pmol of each forward and reverse primer, 0.4 *μ*L of ROX dye, and DEPC-treated water to a final volume of 20 *μ*L. The thermal cycling program for the reactions was as follows: an initial heating for 10 min at 95°C, followed by 40 cycles of 30 sec at 95°C, and 1 min at the annealing temperature of the selected gene. The annealing temperatures of the designed primers are presented in Table 1. Melting curve analysis was performed by increasing the temperature from 65°C to 95°C in 0.1°C/second increments for each fluorescence reading. The efficiencies of the reactions amplifying the FOXO3, MYD88 variants, GAPDH, and beta actin genes were calculated using a standard curve that consisted of 5 points from 10-fold serial dilutions that were prepared using 100 ng of the synthesized cDNA as the first point. Expression levels of the FOXO3, MYD88 variants, and GAPDH genes were normalized using the beta actin gene. The relative expression level for each gene was calculated using REST 2008 software V2.0.7 (Corbett Research, Sydney, Australia).

### 2.8. Detection of Autophagy

Autophagy was detected using real-time PCR to obtain a relative quantification of the Beclin1, PIK3C3, ATG12, LC3, and Gabarapl1 genes in cancerous and normal tissues. The primers were designed using PrimerSelect software version 7.1.0 (Lasergene) and synthesized by the TAG Company (TAG Copenhagen). The real-time PCR reactions were done as described above. The expression levels of Beclin1, PIK3C3, ATG12, LC3, and Gabarapl1 were normalized using beta actin, and relative expression was calculated using REST 2008 software V2.0.7 (Corbett Research).

### 2.9. Statistical Analysis

The *t*-test was used for statistical analysis. Spearman's Rho analysis was applied to assess a possible relationship between the expression levels of the identified differentially expressed genes (FOXO3, GAPDH, and MYD88) and the autophagy-related genes in cancerous and normal tissues. The statistical significance was set at *P* < 0.05.

## 3. Results

### 3.1. Suppression Subtractive Hybridization and Subtraction Efficiency

**Figure 2:**
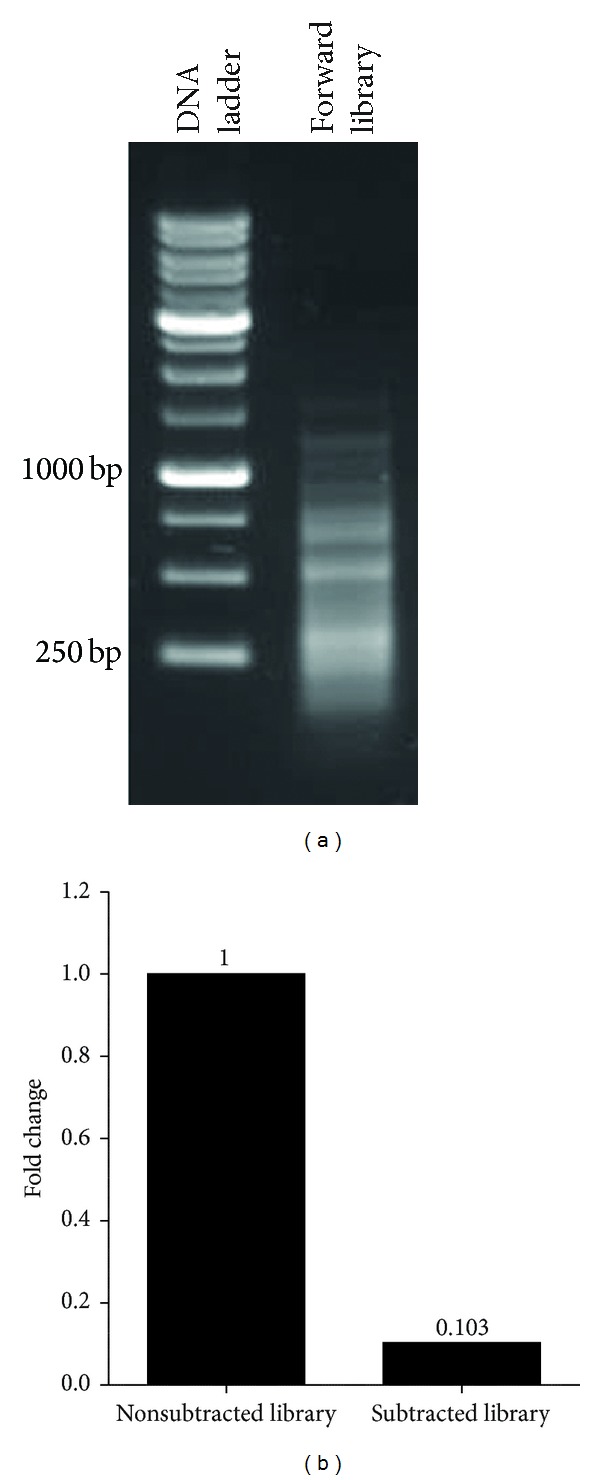
Construction of the SSH library. (a) Forward constructed library (Lane 2), which ranges in length from 180 to 1200 bp. (b) Evaluation of the subtraction efficiency of the constructed library. The relative expression levels of the beta actin gene in the two samples indicate that there is a 9.7-fold decrease in beta actin expression in the subtracted cDNAs.

To identify genes overexpressed in esophageal cancer, SSH was performed using cancerous tissue as the Tester and normal tissue as the Driver.  Figure 2 represents the constructed SSH library, in which the subtracted genes can be seen in the 180–1200 bp region. Among the significantly overexpressed genes (unpublished data), 3 clones, representing the FOXO3, MYD88, and GAPDH genes, were selected because of their probable role in the induction of autophagy. Selected clones were annotated as FOXO3, with a length of 307 bp and 95% identity with FOXO3 sequences, MYD88 with a length of 256 bp and 99% identity with MYD88 sequences, and GAPDH with a length of 758 bp and 98% identity with GAPDH sequences from the NCBI database. Real-time PCR analysis demonstrated that beta actin has a 9.7-fold reduction in the subtracted library compared with the nonsubtracted library.

### 3.2. FOXO3, GAPDH, and MYD88 Are Overexpressed in Esophageal Cancer Tissues

**Figure 3:**
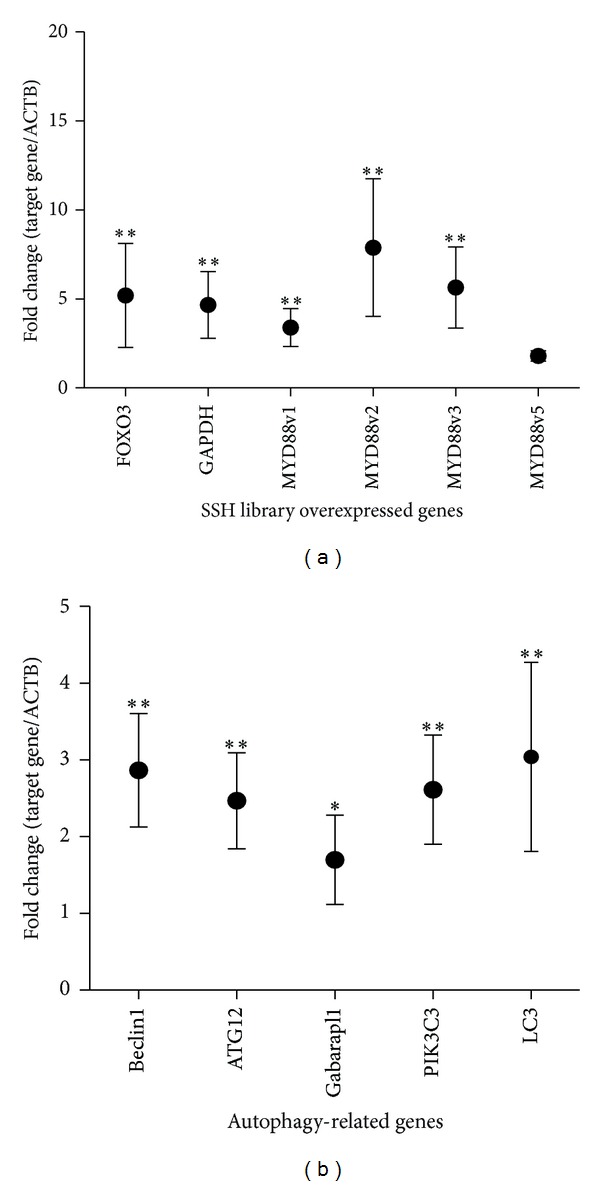
Relative expression of genes identified from the SSH library, MYD88 isoforms- and autophagy-related genes in esophageal cancer tissues, assessed using real-time PCR. (a) Relative expression levels of genes that are overexpressed in esophageal tumor (FOXO3, GAPDH, and MYD88 variants). Expression of MYD88v4 was not detectable in samples. (b) Detection of autophagy in esophageal tumors. Relative expression of autophagy-related genes (Beclin1, ATG12, Gabarapl1, LC3, and PIK3C3) in esophageal tumor tissues. (●) represent the mean and whiskers the SEM of expression in samples (*n* = 10). *P < 0.05, **P < 0.01.

To confirm the results obtained from the constructed SSH library, the expression levels of the FOXO3, MYD88, and GAPDH genes were quantified in cancerous and normal esophageal tissues using qRT-PCR. Overexpression of FOXO3, GAPDH, and MYD88, variants is depicted in Figure 3. These results confirmed the efficiency of the SSH library, which indicated that these genes were significantly overexpressed in esophageal cancer.

### 3.3. MYD88v1 and MYD88v3 Are the Dominant Variants in Esophageal Cancer Tissues

**Figure 4:**
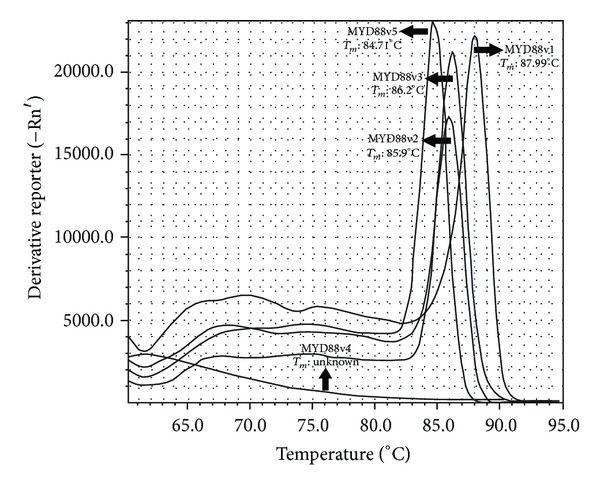
Melting curve analysis of MYD88 variants. Peaks show each variant and its melting temperature in °C. Expression of MYD88v4 was not detectable.

The MYD88 variants were amplified using the Step-One-Plus apparatus. The melting temperatures of MYD88 v1, v2, v3, and v5 were 87.99°C, 85.9°C, 86.2°C, and 84.71°C, respectively (Figure 4). The mean fold induction for each MYD88 variant is depicted in Figure 3. The results demonstrated a significant shift in the pattern of expression of MYD88 variants 1 and 3 in the esophageal cancer tissues compared with the normal tissues. In normal tissues, the expression patterns of the MYD88 variants were as follows: MYD88v1, 10%; MYD88v2, 12%; MYD88v3, 76%; and MYD88v5, 2%. In contrast, the following expression patterns were observed in cancerous tissues: MYD88v1, 25%; MYD88v2, 13%; MYD88v3, 58%; and MYD88v5, 4%.

### 3.4. Overexpression of Beclin1, ATG12, Gabarapl1, LC3, and PIK3C3 Indicates Autophagy in Esophageal Cancer Tissues

The transcript level of Beclin1, a gene that is involved in autophagic vacuole formation and acts as a coregulator of autophagy increased 3.825-fold in tumor tissues. The transcript levels of LC3, ATG12, and Gabarapl1 genes involved in autophagic vacuole formation, increased 3.04-fold, 2.685-fold, and 1.820-fold in tumor tissues, respectively. The transcript level of PIK3C3, a gene that acts as a coregulator of autophagy and is involved in the induction of autophagy in response to intracellular signals, was 2.704-fold higher in tumor tissues than in normal tissues (Figure 3). Correlation of FOXO3, MYD88 isoforms, and GAPDH with autophagy-related genes is represented in Table 2.

## 4. Discussion 

Autophagy is categorized as a protein degradation system, which participates in the cellular homeostasis that is typically observed in nutrient-deprived cells. In this process, excess or unnecessary proteins and organelles are fused to lysosomes and digested by lysosomal enzymes [11, 12]. In the early stages of the tumorigenesis and during cancer treatment, autophagy induction fortifies cancer cell growth in the tumor microenvironment by protecting cells from nutrient deprivation and providing an alternative energy source for cancer cells [13, 14].

In this study, suppression subtractive hybridization was used to identify the overexpressed genes, FOXO3, MYD88, and GAPDH, which have potential roles in the induction of autophagy in esophageal cancer cells. Upregulation of these genes was confirmed in esophageal cancer samples using qRT-PCR.

The work presented here identified the FOXO3 gene as being overexpressed in esophageal cancer. This gene belongs to a family of transcription proteins that is involved in the regulation of autophagy. Autophagy-related genes (e.g., LC3 and BNIP3) are directly upregulated by FOXO3 [15, 16]. In addition, previous studies revealed that FOXO3 overexpression induced autophagy in the HEK293T cell line [17]. Our results indicated that FOXO3 was upregulated 5.187-fold in esophageal cancer tissues. FOXO3 overexpression in cancer cells could be related to nutrient deprivation stress and other stimulatory factors that lead to autophagy induction. Elevated expression of the transcription factor FOXO3 induces autophagy protein expression and promotes the induction of autophagy.

MYD88 is another overexpressed gene in the constructed library. MYD88 is a cytosolic adaptor protein in the interleukin-1 (IL-1) and Toll-like receptor (TLR) signaling pathways, which have critical roles in innate and adaptive immunity [18]. Previous studies demonstrated that MYD88 has an important function in tumorigenesis, and high levels of MYD88 correlate with poor prognosis in patients with colorectal cancers [19]. Lipopolysaccharides, a TLR receptor ligand, upregulates *β*-1 integrin, which leads to tumor cell-endothelial cell adhesion, tumor cell-extracellular matrix adhesion, and tumor cell invasion of the extracellular matrix [20]. In addition, MYD88 promotes tumor cell proliferation via NF-*κ*B activation. Previous studies demonstrated a direct interaction between MYD88 and Beclin1. Interaction of MYD88 with Beclin1 reduces Beclin1 binding to Bcl-2, which leads to autophagy induction in cancer cells [21]. Our results revealed for the first time that MYD88 variants are upregulated in esophageal cancer tissues compared with normal tissues. Our findings indicated that MYD88 overexpression could lead to autophagy in esophageal cancer. The cause of MYD88 overexpression in esophageal cancer tissues likely originates from the extracellular tumor matrix, where the presence of mucin could trigger prolonged activation of TLR receptors and subsequent upregulation of MYD88. Compared with normal esophageal tissues, there was a significant reduction in MYD88v3 levels and a significant increase in MYD88v1 levels in esophageal tumor tissues. Interaction of MYD88 with Beclin1 has been previously demonstrated, but no information is available concerning the participation of individual exons in this interaction; thus, the role of each of the MYD88 variants in the interaction with Beclin1 and the subsequent induction of autophagy remains to be elucidated.

GAPDH is a key protein in the production of energy. Overexpression of GAPDH has been reported in cancers, and the primary role for GAPDH was proposed to be its pivotal function in glycolysis. However, cooperation of GAPDH with ATG12 could induce autophagy instead of caspase-independent cell death (CICD) [22]. After apoptotic cytochrome c release without caspase activation, which leads to CICD, GAPDH elevates ATP levels in the cells by inducing glycolysis and cooperates with ATG12 to shift the cells away from CICD and toward autophagy. In our SSH library, GAPDH was identified as an overexpressed gene, with a 4.659-fold induction. Nutrient deprivation, hypoxia, and high levels of cell division demand additional energy resources, and GAPDH overexpression could alleviate these demands by inducing both glycolysis and autophagy in esophageal cancer cells.

Because this is the first report of the overexpression of the FOXO3, GAPDH, and MYD88 genes in esophageal cancer cells, the precise role of these genes in esophageal cancer remains to be elucidated. Significant correlations between the expression levels of these genes and those of autophagy-related genes point to a potential role for these genes in the induction of autophagy. Prolonged autophagy induction in tumor tissues and degradation of autophagy-related proteins via autophagosome formation leads to the upregulation of autophagy-related genes in esophageal cancer tissues. FOXO3 overexpression correlated with expression of the Gabarapl1, ATG12, PIK3C3, LC3, and Beclin1 genes in cancer tissues. In light of its transcriptional activity and regulatory effects on the process of autophagy in skeletal muscle, FOXO3 could increase the expression level of autophagy-related genes in esophageal cancer tissues [15]. A significant correlation was observed between the expression of GAPDH and the autophagy-related genes ATG12 and PIK3C3 in the tissue samples. This correlation could be related to the autophagy-induction activity of GAPDH, which causes a subsequent upregulation of autophagy-related genes. Overexpression of MYD88v1, MYD88v2, and MYD88v3 correlated with upregulation of the autophagy-related genes Beclin1, ATG12, LC3, and PIK3C3. This correlation could be related to the induction of autophagy via TLR receptors, which leads to MYD88 upregulation.

The results of this study suggest that the induction of autophagy in cancerous tissues is an important event in the molecular biology and tumorigenesis of esophageal cancer. Tumor cells experience metabolic stress and starvation caused by increased proliferation and cell metabolism and hypoxia caused by inadequate angiogenesis, in which ATP production is inefficient. These environmental stresses induce autophagy in tumor cells located far from the blood vessels [23]. Autophagy decreases the energy consumption of tumor cells by reducing metabolic stress and eliminating cellular organelles that limit damage. Autophagy has previously been studied as a target for the development of new therapies for esophageal cancer because inhibition of autophagy has the potential to sensitize tumor cells, including chemoresistant cells, to therapeutic agents [24]. To date, autophagy has not been considered a fundamental molecular event in esophageal cancer, and the role of autophagy in the tumorigenesis of this cancer has not been addressed. Therefore, future studies that focus on autophagy as a target for cancer therapeutics are necessary. Because of intraepithelial neoplasia importance in tumorigenesis of esophageal cancer, occurrence of autophagy and expression of FOXO3, MYD88, and GAPDH genes should be studied in this stage. This could reveal the role of the above genes in the early stage of tumorigenesis.

In conclusion, using the SSH method, our study revealed for the first time that the FOXO3, MYD88, and GAPDH genes are overexpressed in esophageal cancer. In addition, there was a significant correlation between overexpression of these genes and upregulation of autophagy-related genes in esophageal cancer. Considering the fundamental role of autophagy in tumorigenesis and the high expression levels of FOXO3, MYD88 and GAPDH in esophageal cancer, further studies are needed to evaluate the roles of these genes and autophagy in the development of esophageal cancer. In addition, autophagy could be responsible for the poor prognosis and chemoresistance that are observed in esophageal cancer patients. The overexpression of the FOXO3, MYD88, and GAPDH genes could lead to the induction of autophagy genes in esophageal cancer. Thus, these genes are candidates for targeting autophagy and tumorigenesis in esophageal cancer.

## Figures and Tables

**Table 1:** Designed primers sequences used to quantify gene expression by real-time PCR.

Application	Primer name	Accession number	Length	Sequence (5′ to 3′)	Annealing temperature	Location	Product size
Overexpressed genes	FOXO3-F	NM_001455.3	20	ACGGTGTTCGGACCTTCATC	60°C	1613–1632	196 bp
	FOXO3-R	NM_001455.3	20	TGCTGGCCTGAGACATCAAG		1789–1808	
	GAPDH-F	NM_002046.4	20	CGACAGTCAGCCGCATCTTC	61°C	118–137	250 bp
	GAPDH-R	NM_002046.4	20	CGTTCTCAGCCTTGACGGTG		348–367	
	MYD88-v1-F	NM_001172567.1	20	TCATCGAAAAGAGGTTGGCT	57°C	855–875	158 bp
	MYD88-v1-R	NM_001172567.1	20	GATGGGGATCAGTCGCTTCT		993–1012	
	MYD88-v2-F	NM_002468.4	20	CCACACTTGATGACCCCCTG	61°C	666–685	210 bp
	MYD88-v2-R	NM_002468.4	20	GGCGGCACCTCTTTTCGATG		856–875	
	MYD88-v3-F	NM_001172568.1	20	GGGACCCAGCATTGGGCATA	61°C	538–557	289 bp
	MYD88-v3-R	NM_001172568.1	20	ACCTGGAGAGAGGCTGAGTG		807–826	
	MYD88-v4-F	NM_001172569.1	20	CTTGATGACCCCCTGGGTGC	62°C	671–690	207 bp
	MYD88-v4-R	NM_001172569.1	20	GGTTGGTGTAGTCGCAGACA		858–877	
	MYD88-v5-F	NM_001172566.1	17	ACCCAGCATTGGTGCCG	60°C	541–557	202 bp
	MYD88-v5-R	NM_001172566.1	20	GGTTGGTGTAGTCGCAGACA		723–742	

Control gene	ACTB-F	NM_001101.3	21	ATGGCCACGGCTGCTTCCAGC	60°C	763–783	322 bp
	ACTB-R	NM_001101.3	21	CAGGAGGAGCAATGATCTTGA		1064–1084	

Autophagy-related genes	Gabarapl1-F	NM_031412.2	20	CAGGGTCCCCGTGATTGTAG	61°C	321–340	179 bp
	Gabarapl1-R	NM_031412.2	20	TGGGAGGGATGGTGTTGTTG		480–499	
	Beclin1-F	NM_003766.3	20	GCTGAAGACAGAGCGATGGT	59.5°C	30–49	169 bp
	Beclin1-R	NM_003766.3	20	CATGGTGCTGTTGTTGGACG		179–198	
	PIK3C3-F	NM_002647.2	20	GGCACACAGAGTGAGCAGTA	59.5°C	2423–2442	204 bp
	PIK3C3-R	NM_002647.2	20	CACAGCCTCTTCATCCGACA		2607–2626	
	ATG12-F	NM_004707.3	20	TGCTGGAGGGGAAGGACTTA	59.5°C	347–366	186 bp
	ATG12-R	NM_004707.3	20	GTTCGCTCTACTGCCCACTT		513–532	
	MAP1LC3B-F	NM_022818.4	20	GCCGCCTTTTTGGGTAGAAG	59.5°C	1012–1031	225 bp
	MAP1LC3B-F	NM_022818.4	20	TACCCCTGCAAGAGTGAGGA		1217–1236	

**Table 2:** Correlations of FOXO3, MYD88 isoforms, and GAPDH with autophagy-related genes.

	LC3	Gabarapl1	ATG12	PIK3C3	Beclin1
FOXO3	0.000	0.007	0.007	0.000	0.000
MYD88v1	0.000	0.500	0.007	0.007	0.000
MYD88v2	0.000	0.000	0.000	0.000	0.000
MYD88v3	0.000	0.062	0.000	0.007	0.000
MYD88v5	0.062	0.062	0.062	0.062	0.229
GAPDH	0.062	0.500	0.000	0.000	0.062

Correlations were considered significant when *P* < 0.01.
